# III persons and capable workers: Constructing work ability in return-to-work negotiations after sickness absence

**DOI:** 10.1177/13634593241290176

**Published:** 2024-10-14

**Authors:** Hanna Keränen, Sanni Tiitinen, Pirjo Juvonen-Posti, Elina Weiste, Soile Seppänen, Leena Ala-Mursula

**Affiliations:** University of Oulu, Finland; Finnish Institute of Occupational Health, Finland; Jyväskylä University of Applied Sciences, Finland; Finnish Institute of Occupational Health, Finland; Finnish Institute of Occupational Health, Finland; Finnish Institute of Occupational Health, Finland; University of Oulu, Finland; University of Oulu, Finland

**Keywords:** conversation analysis, membership categorization analysis, return-to-work, shared decision-making, work ability

## Abstract

In return-to-work (RTW) negotiations after sickness absence, the work ability of an individual employee becomes a shared interest for the multiple stakeholders representing both the healthcare sector and working life. In practice, the employee, employer and occupational health professionals need to reach a shared understanding of the employee’s work ability to enable shared decision-making concerning the plans for sustainable RTW. Drawing on 14 video-recorded RTW negotiations, we used conversation analysis-informed membership categorization analysis to examine how the participants of RTW negotiations discuss the work ability of an employee to pursue a shared understanding of the situation. Work ability was constructed in a very situational way, using illness categories to both explain the work ability of the employee and argue for or against their ability or inability to work. Our study contributes to research on RTW by introducing a new perspective to work ability. We show how work ability is realized during RTW negotiations through interaction, and how participants leverage their cultural understanding of illness and capability when negotiating work ability. We also demonstrate how membership categorization analysis can reveal the situational and consequential aspects of illness and work ability categories.

## Introduction

During the planning of return to work (RTW) after sickness absence, the work ability of an individual employee becomes a shared concern for multiple stakeholders in healthcare, rehabilitation, and working life. To achieve sustainable RTW for the employee, these stakeholders need to communicate closely with each other to reach a shared understanding of the employee’s situation as well as any possibly required work modifications (Burstrom et al., 2011; Dasinger et al., 2001; Soklaridis et al., 2010). However, little is known about multi-stakeholder communications related to RTW.

Important arenas for these communications are RTW negotiations among employees, employer representatives, and occupational healthcare professionals (Lappalainen et al., 2019). The goal of an RTW negotiation is to make shared decisions concerning the employee’s future RTW. RTW negotiations are effective: work participation can be enhanced (Lappalainen et al., 2019) and sickness absences shortened after shared decisions regarding work modifications are made in RTW negotiations (Reho et al., 2018). Moreover, a multistakeholder meeting of different rehabilitation actors about employees on long-term sickness absence has shown to increase the probability of initiating occupational rehabilitation measures (Burstrom et al., 2011).

In RTW negotiations, shared decisions are made by the doctor, employee and employer (Ristimäki et al., 2020). In the Finnish healthcare system, the context of our study, the employee is often a patient of the occupational health physician, making RTW negotiations an event during which healthcare, working life, and individual views intersect. Shared decision-making (SDM) has been widely studied in doctor–patient health encounters (Coulter and Collins, 2011; Land et al., 2017), but less so in health-related multiparty encounters. However, some studies have focused on rehabilitation encounters and healthcare interactions involving patients’ companions (Barnard et al., 2010; Keel and Schoeb, 2017; Pino et al., 2021).

In this study, we examined how the RTW negotiation participants construct and negotiate the employee’s work ability before moving toward shared decision-making about the employee’s future. Previous studies have shown that all communication during healthcare encounters is important for SDM (Land et al., 2017) and that SDM extends beyond the actual moments of decision-making in healthcare encounters (Matthias et al., 2013).

Nevertheless, a shared understanding is not a self-evident outcome of discussions in RTW negotiations, as the participants bring potentially different views to the negotiation. First, the participants’ interests and viewpoints are shaped by the contexts from which they come (Ståhl et al., 2010). Occupational health professionals may perceive encouraging RTW as a meaningful task, for both enhancing the employee’s well-being and reducing disability costs for society (Horppu et al., 2016). Representing the workplace perspective, employers may emphasize the organization’s productivity or economic limitations (Seing et al., 2012). For employees, the perceived meanings of their disabilities and the individual relevancy of RTW are crucial (Shaw et al., 2002).

Second, the participants may have different views on work ability, as its definition has evolved over the decades. Initially, work disability was considered to be the result of biological pathology and thus objectively observable and correctable (Schultz et al., 2007). Over time, the concept broadened, as it was understood that illness does not always equal work disability and that non-medical causes may explain long-term work disability (Loisel, 2009). However, in the social insurance context, earlier views assuming a linear causation between physical impairment and work disability, or considering it the result of the interaction between health and work requirements, still prevail (OECD, 2010).

In vocational rehabilitation and RTW processes, there has been a shift from disability paradigms to a demedicalizing ability paradigm, which emphasizes the individual’s abilities and opportunities instead of their disabilities and limitations (van Hal et al., 2013). The goals are that employees can continue working despite health issues and that employers modify their work tasks accordingly (Lefever et al., 2018). The ability paradigm views work ability as multidimensional, dynamic and influenced by the social environment (Ilmarinen, 2009). Recent conceptualizations also consider work (dis)ability a lifelong process, involving many factors such as personal resources and the work environment, and varying across different contexts (Lederer et al., 2014).

In sum, various views and conceptualizations of work ability exist, with illness plays different roles. Our study posits that these different conceptualizations are part of the cultural knowledge on work ability that the participants use as a resource in RTW negotiations. This occurs through *categories* which the participants invoke and utilize during their discussions (Sacks, 1992). We applied membership categorization analysis (MCA), which posits that people constantly classify knowledge about themselves, others, and the world through categories (Sacks, 1992; Silverman, 1998). Cultural knowledge is used for categorizing, and culture is constituted through social actions (Hester and Eglin, 1997). MCA allowed us to study the participants’ own constructions and negotiations of work ability, as they talked it “into being” (see Heritage and Clayman, 2010).

A person can always be categorized in multiple ways (Hauser, 2011; Sacks, 1992). For example, the same employee in an RTW negotiation may be categorized as a multimorbid patient incapable of working or as a person with illness-related limitations but young enough to still have career options. The selection of categories is significant, as the chosen categories and their recognition by the recipients influence their consequences (Goodman and Speer, 2007; Stokoe, 2012). Thus, the categories used in discussions of an employee’s work ability in RTW negotiations can affect the decisions made about the employee’s future. As Housley and Fitzgerald (2009) note, applying MCA allows the study to view culture as an action through which social and moral order is accomplished and negotiated.

However, what remains unstudied is how this happens in practice, that is, how the RTW negotiation participants construct the meaning of work ability when negotiating the employee’s situation before making shared decisions. We studied this question using recordings of authentic RTW negotiations as data. More precisely, we investigated the following research questions: (1) How do the RTW negotiation participants construct and use categories of illness and work ability when discussing the employee’s RTW and (2) what is achieved in the discussions by using these categories? Specifically, we were interested in the relevance of illness categories for explaining or arguing for or against an employee’s ability or inability to work.

## Materials and methods

Our data consisted of 14 video-recorded and naturally occurring RTW negotiations, collected in a joint research project Finnish Institute of Occupational Health and Tampere University. All the negotiations included an occupational health physician, an employee, and one or two supervisors. In 12 cases, one or two of the following participants were also present: an occupational health nurse, a human resources professional, an occupational safety representative, an occupational physiotherapist, and a physician specializing in occupational health.

All the participants gave their written informed consent, and the study was approved by the Ethics Committee of Finnish Institute of Occupational Health. Two video cameras placed on opposite sides of the room recorded the sessions. The negotiations lasted between 25 and 60 minutes, totaling 540 minutes of recordings.

We analyzed our data using a type of MCA best described as conversation analysis-informed (CA-informed) MCA. As a qualitative and empirical method for examining categorial or topical issues, MCA can be applied to various types of data, including recordings of interactions, interviews and texts, with different emphases (Speer and Stokoe, 2011; Stokoe, 2012). As the focus of CA is the structural analysis of conversation addressing sequential matters such as the organization of turns-of-talk and the ways in which people design these turns (see Sidnell and Stivers, 2013), combining this and MCA enabled us to address both the constructed reality and the communicative practices (Stokoe, 2012) when pursuing a shared understanding of an employee’s work ability in RTW negotiations. As categories come alive in and are recognized during interaction, sequential matters are an important part of the analysis. Thus, with CA-informed MCA, the sequential and categorial aspects of social interaction inform each other in the analysis, as category work is embedded in the sequential organization of conversation (Silverman, 2012).

We followed the analytical process proposed by Stokoe (2012). The first author built a collection of sequences in which the participants constructed the employee’s work ability using descriptions of the employee’s illnesses. We included descriptions resonant with the categories of being ill (e.g. “gone through surgery,” “suffering from depression”) and being capable of working (e.g. “able to use cleaning machines,” “able to work a whole day”). We paid close attention to both the explicit and implicit categorizations associated with, for example, activities and attributes (see Widdicombe, 2008).

The first author then transcribed the sequences according to CA conventions (Jefferson, 2004). Next, the first author analyzed each sequence and discussed them with the second author. We located the sequential positions of the categorial instances within the ongoing interaction and analyzed the design and action orientation of these sequences. We examined how the participants orientated toward the categories: how the categories of being ill were used and negotiated with respect to the categories of being capable of working. After analyzing the data extracts case by case, we compared them, to identify patterns in how the participants used, constructed, and oriented toward the categories of being ill when discussing work ability. (See Sidnell, 2020; Stokoe, 2012.)

In the Results section, we demonstrate our findings using three data extracts that most clearly illustrate the different ways of constructing and using categories across the entire dataset.

## Results

In this section, we show three ways in which the participants use the categories of being ill in their discussions on work ability. First, we illustrate how the category of an ill person is used and accepted as a background and a starting point for negotiating work ability, and in this case, also work accommodations (Excerpt 1). Second, we demonstrate how non-membership of the ill person category is used to reassure the participants of the employee’s work ability (Excerpt 2). Third, we show how the participants construct opposing meanings for the ill person category when arguing for or against work ability, leading to different views concerning the employee’s situation and the plans for RTW, and to interaction (Excerpt 3).

### Using ill person category as a background

Excerpt 1 shows how ill person category is used and accepted as a jointly understood background for the negotiation, thereby entitling the employee to work modifications. The participants are planning the employee’s RTW after her back operation. Before this excerpt, the doctor has asked the employee to describe her situation, and the participants have discussed the nature of her job (EE = employee, SV = supervisor, DR = doctor).

**Excerpt 1.**

**1 EE:**  sit et miten (.) mikä on niinko yleensä [tällasessa

     **and so how (.) what is like usually [in this kind of**

**2 SV:** **                           [mm-m**

**3 EE:**  tapauksessa sie varmaan oot näitten (.) selkäihmisten kanssa

     **situation you’re probably more familiar with these (.) back**

**4**     [ollut

     been

     **people**

**5 SV:**   [nii

     **[yeah**

**6 EE:**     enemmänki  tekemisissä että (.) minkälaine (.) työ niinku

     even+more involved     so what+kind+of work like

     **so (.) what kind of work from a**

**7**       lääkärin näkökannasta .hh

     **doctor’s point of view .hh**

**8 SV:**     nii

     **yeah**

**9 DR:   [mm**

**10 EE:**  [ois (.) parempaa ku se (.) pakatun tekem[inenki

     **[would be (.) better because(.) packing (food)**

**11 SV:** **                          [mm ((nods))**

**12 EE:**   on sitä että siin ollaan hirveen paljon paikallaan että (.)

     is that that there one+stays awfully lot still so

     **means that you just stay still an awful lot so (.)**

**13** [seisotaan vaan

     **[you just stand**

**14 SV:** [mm nii

      **[mm yeah**

**15 DR:  ((nodding))**

**16 EE:**    ja  esimerkiks ku [pakataan noita (0.8)

     **and for example when [you pack those (0.8)**

**17 SV:** **              [mm-m**

**18 EE:**   esimerkiks (.) ananas porkkanaa [semmosii pienii

     **for example (.) pineapples and carrots [into those little**

**19 SV:**                          [nii

            **                [yeah**

**20 EE:**    rasioihin niinku ((tekee käsillä kauhomisliikettä))

     **boxes like ((scoops with her hands))**

**21 SV:   mm-m**

**22 EE:**   pitkän aikaa on vaa tällee,

     **for a long time you just stay like this**

**23 SV:**  nii-i

     **yeah**

**24 EE:**  paikallaa.

    **still.**

**25 SV:   mm-m**

**26 EE:**   et mikä sit ois niinko

     **so what then would be like**

**27 SV:**  [nii mikä sit selän kannalta nyt [olis sitte

     **[yeah what would be then considering the back**

**28 DR:**  [.hh                   [hh täydellisessä maailmassa

     **[.hh                   [hh in a perfect world you**

29      olisi liikkuva työ jossa olisi niinku kevyttä nosteltavaa –

      **would do mobile work that would like include light lifting –**

      ((8 lines omitted as DR continues describing a mobile job and then states that a perfect job does not exist))

37 DR:   mut et jos aattelee nii aamupakatussa

       **but if we think about the morning shift with packing food**

38      toisaalta niinku ois työnkuva  ehkä >paras<

       on+one+hand like would+be job+description maybe best

       **on the one hand would probably have the best job description**

39    mut sitte taas et ku niit on vaa yks ihminen tekemässä

       but then again that those are only one person doing

       **but then on the other hand only one person works the shift**

40      toki se [että loma-

        **although the fact [that vaca-**

41 EE:             [mm pitää muutki ottaa huomioon

                 **[mm one must consider others too.** ((nodding))

The ill person category is invoked in the employee’s question to the doctor (starting in line 1) when she uses the expression “back people” (line 3), which can be interpreted as “people with a back condition” on the basis of earlier discussions. The employee’s question is designed in a way (“you’re probably more familiar,” line 3, “from a doctor’s point of view,” lines 6–7) that acknowledges the doctor as an epistemic authority in medical questions (see Stivers et al., 2011). The employee constructs herself as an ill person with a back condition, placing herself in the “back people” category. The use of an unconventional expression and indirect reference indicates that the employee treats the category as jointly understandable.

The supervisor and the doctor treat the category constructed by the employee as recognizable. The supervisor confirms it with “yeah” (line 5) immediately after the employee mentions “back people.” She aligns with the employee’s question concerning a suitable job for “back people” (“yeah”, line 8) and later repeats the question (line 27), indicating that she treats the question of suitable work tasks for “back people” as medical. The doctor responds to the employee’s question (lines 28–29) with a turn designed to be an answer and not, for example, a request to clarify the category embedded in the employee’s question, indicating her understanding of it. All the participants thereby orient toward a shared understanding of what the expression “back people” means and acknowledge the employee’s membership of the ill person category of people with a back condition.

Simultaneously, the participants treat this category membership as something that does not completely prevent the employee from working. The employee constructs her incapability using examples of the work tasks that are problematic for her: packing food (line 10) and standing still for long periods (lines 12–13). She emphasizes these difficulties using the exclusive particle “just” (lines 12 and 22), the comparative form “better” (line 10) and the extreme case formulation “an awful lot” (line 12). Extreme case formulations can be used to legitimize claims in interaction (Pomerantz, 1986). Thus, the employee implies that “back people” are not members of the capable worker category with respect to the work tasks discussed here. However, by asking for the doctor’s opinion of a more suitable job, she implies that she is not incapable of all types of work. The supervisor aligns with this by repeating the employee’s question about suitable work tasks (line 27).

The doctor’s response (from line 28 onward) indicates that she also recognizes the employee’s inability to perform the described tasks, but not all types of work. By describing perfectly fitting tasks in an imaginary world, (from line 29 onward), the doctor acknowledges the unsuitability of the current tasks, thus partly excluding the employee from the capable worker category. This description serves as pre-proposal work (see Arminen, 2005), before the doctor continues by suggesting the morning shift with packing food (lines 37–38) but expresses uncertainty about its feasibility (lines 39–40). The employee confirms this uncertainty (line 41).

This excerpt shows that all the participants treat the employee as a member of the ill person category, making her incapable of some work tasks. However, this incapability is not seen as total, and the employee is considered to be entitled to work modifications to support her remaining work ability. The employee’s illness serves as a jointly accepted background and a starting point for negotiating work ability and any necessary modifications.

### Reassuring the participants of the employee’s ability to work

Excerpt 2 demonstrates how non-membership in the ill person category is used to reassure the participants of the employee’s ability to work. This excerpt is from a negotiation in which the participants are planning the employee’s RTW by trying to find a new role for him within the organization. Earlier in the negotiation, the employee’s incapability to perform his current work as a driver due to a mental health condition, was jointly acknowledged and seen as a starting point, like in Excerpt 1. The employee has strongly expressed a desire to work again, and the career counselor has proposed a janitor’s job. We join the excerpt as the career counselor is elaborating on the proposal (CC = career counselor, EE = employee, DR = doctor, SV = supervisor).

**Excerpt 2.**

**1 CC:**  joo et niitä↑ki     o erilaisia    riippuen sitte että

     **yeah so there are different kinds of them depending on**

**2**      koulujen .hh vahtimestarit niin siinä on sitte sitä fyysistä

     . **hh so a janitor’s work in schools includes physical**

**3**      työtä mut sullahan ei oo    mitään fyysistä rajotetta.

     **work but you don’t have any physical limitations, do you. ((looks at EE))**

**4 EE:**     ei o         [päinvastoin

     **no I don’t [on the contrary**

**5 CC:**                     [eli siellä sitte .hh on tota jonku verra sitte

                    **[so that .hh would include like to some extent**

**6**     sellasia pieniä korjaus .hh huoltohommia ja (.) tietysti

     **some small repairing and maintenance tasks and of course**

**7**      varmaan .hh [semmosta tavaroiden siirtoa ja,

     **probably .hh [sort of moving things and**,

**8 DR:**                        [tavaroiden siirtelyä ja kaikkee tämmöstä.

                        **[shifting things and things like that.**

**9 EE:  [mm-m ((nodding))**

**10 CC:**    [tämmöstä että (0.2) tota niin (0.4) on sellanen aika (0.6)

     **[like that so (0.2) erm so (0.4) one is sort of quite (0.6)**

**11**     monipuolinen vähä semmonen joka paikan (.) höylä sitte siellä

     **a diverse a little bit of a jack of all (.) trades then there**

**12**   (0.3) koululla

    **(0.3) at the school**

**13 DR:**  mut se on sillai     hyvä ku    ei    oo mitään selkävikaa

     **but it’s like good that you don’t have any back problems**

**14**     niskavikaa,

     **neck problems**,

**15 CC:** [joo

     **[yeah**

**16 DR:** [käsiv[ikaa     [jalkavikaa

     **[arm problems [leg problems**

**17 EE:**                          [ei ((pudistelee päätään))

**                        [no ((shaking his head))**

**18 SV:                          [((nodding))**

**19 DR:**   et [sillai

     **so [like**

**20 EE:**       [ei ((pudistelee päätään))

      **[no ((shaking his head))**

**21 DR:**  ei oo                [niinku periaatteessa

     **you don’t have [like in principle**

**22 SV:**                          [ei oo mitään rajotteita [siihen puoleen

                           **[you have no limitations [insofar as**

The career counselor and the employee jointly categorize the employee as a capable worker for the janitor’s job. The career counselor describes the physical requirements but treats the employee as capable by asking for confirmation that he has no physical limitations (lines 2–3). Her turn is designed as a negative declarative, preferring a negative answer (Heritage, 2010), implying that she considers the lack of physical limitations a shared fact. Thus, she seeks confirmation of the employee’s capability rather than discussing any illness that might cause physical limitations. The employee accepts this categorization by responding as preferred (“no I don’t,” line 4), and further confirms his lack of physical limitations with “on the contrary” (line 4). This constructs him as very physically capable and distances him from someone with physical impairments. The career counselor continues to describe the job immediately after the employee’s “no I don’t,” focusing on the work tasks (lines 5–7 and 10–12). This indicates that she considers the issue of physical capability resolved.

The doctor responds to the career counselor’s description of the physical nature of the job by invoking the ill person category (from line 13 onward). He uses the category to reassure the participants, including the employee, of the employee’s capability to perform the job and does this by constructing the employee’s non-membership in the category of physically ill persons. He contradicts the possibility that the employee is incapable of performing the physical tasks (“but it’s like good that,” line 13) and listing in detail the physical problems that the employee does not have (lines 13, 14 and 16) instead of using a more general expression such as “musculoskeletal diseases.” This detailed listing maximizes the lack of physical illnesses as supporting the employee’s capability (see Potter, 1996). The use of “problem” (lines 13, 14, and 16) can be heard as a difficulty or an issue, whereas a term such as “condition” would imply a state. By constructing physical illnesses as problematic and emphasizing the employee’s lack of them, the doctor excludes the employee from the ill person category.

It is notable that the doctor invokes the ill person category and constructs the employee’s exclusion from it even after the career counselor and the employee have concluded that the employee is physically capable, and the discussion has moved on. By further explaining the employee’s capability in terms of the lack of illnesses, the doctor reveals not only his own reasoning, but also his need to convince the other participants, including the employee, of the employee’s capability (see Antaki, 1994). The employee repeatedly responds with “no” (lines 17 and 20), confirming his exclusion from the ill person category. The supervisor aligns with the doctor by nodding (line 18) and making a formulation of the doctor’s speech (line 22). The employee’s repeated negative answers further distance him from someone with physical impairments.

In sum, this excerpt demonstrates how the ill person category, or in this case, situationally constructed non-membership of the category, is used to reassure the negotiation participants of the employee’s capability. Although the discussion between the career counselor and the employee starts from the perspective of the ability to perform work tasks, the doctor invokes and uses the ill person category as part of his interactional work to convince the others of the employee’s work ability.

### Opposing another participant’s argument by constructing a different meaning for the ill person category

Last, we show an example of a case in which the participants construct the ill person category as having different meanings, and in this way, utilize it in to oppose an argument. In Excerpt 3, the participants are planning the employee’s RTW after recurring sickness absences. At the beginning of the negotiation, the employee has said that he wishes to apply for partial disability pension, whereas the doctor has proposed retraining through vocational rehabilitation. The employee has declined this proposal. Now, the participants are discussing other options and making decisions (DR = doctor, SV = supervisor, OSR = occupational safety representative, EE = employee, NU=nurse).

**Excerpt 3.**

**1 DR:**  mm ((nyökkää)) [mutta jos

     **mm ((nods))    [but if**

**2 SV:**              [joo mm

              **[yeah mm**

**3 DR:**    aatellaan ni meiän tämähetkinen .hh keino ehkä selevitä etteenpäin

     **we think about it then perhaps .hh our way to move forward now**

4       olis kuitenki se osasairasloma [että voiaan palata nyt töihin

     **would however be partial sick leave [to enable return to work**

**5 OSR:**                           [nii siitä lähetään etteenpäin

                             **[yeah that’s where we start from**

**6 NU:**     mm [sitten

     **mm [then**

**7 DR:**     [ja sinä aikana voiaan kattoo eläkevakuutusyhtiön kans ne

        **[and during this, with the pension provider, we can look at those**

**8**    ((katsoo työntekijää)) (.) eläk- osa[eläke[päätökset ja

      **((looks at EE)) (.) decisions on par[tial [pension and**

**9 NU:**                         [nii

                             **[yeah**

**10 OSR:                       [mm-m**

**11 NU:**                            **[mm**

**12 DR:**  ja [tiä- tiätkö että so- ((katsoo TT))

     **and you know that yo-** ((looks at EE)

**13 SV:**    [juuri näin

         **[that’s right**

**14 DR:**    sää oot sen ↓ikänen ja (.) perusteena

     **You’re at that age and (.) the grounds ((for the application))**

**15**     on tules-sairauet että (.) e- elä (.) elä ihmettele

     **are musculoskeletal diseases so (.) d- don’t (.) don’t be surprised**

16     jos sieltä tullee semmonen päätös [että ((katsoo työntekijää))

     **if you get a decision in which    [they ((looks at EE))**

**17 NU:                       [mm**

**18 DR:**  ei myönnetä ellä- osaeläkettä mytta mutta myönnetään

     **refuse to grant pen- partial pension but they grant**

**19**     koulutukseen rahat.

     **money for retraining.**

**20 EE:**    [joo

     **[yeah**

**21 OSR:** [joo-o

     **[yea-ah**

**22 NU:**  joo

     **yeah**

**23 DR:**    sieltä tullee nykyään [semmosia (0.3) kirjeitä.

     **they send [these kinds of (0.3) letters nowadays.**

**24 NU:**               [nii se on niinku (.) nii hyvä tietää

                   **[yeah it’s like (.) good to know**

**25**    [että tämmönen on mm

     **[that this is the case mm**

**26 EE:** [no aatellaanpa semmonen tilannekki että (0.5) täällä varmaan

     **[well let’s also think about the situation that (0.5) probably**

**27**     ainoo joka (0.6) Linda ja Laura ymmärtää että o- vuojesta

     **the only ones who (0.6) understand are Linda and Laura that since**

**28**    seiskytkaheksan sairastanu ykköstyypin

     **nineteen seventy-eight I’ve had type one**

**29 DR:**    **mm ((nods, looks at EE))**

**30 EE:**  diabete[sta

     **diabetes**

**31 NU:** **         [mm**

**32 EE:**   huonolla insuliineilla

     **with bad insulin levels**

**33 DR:**   mm ((nyökkää, katsoo TT))

     **mm ((nods, looks at EE))**

**34 EE:**   kaikilla (0.6) kaikki silimänpohjat on mulla operoitu nyt

     **with all (0.6) the fundi of my eyes have been operated on and now**

**35**      mennää uuestaa o- operointii vuojen vaihteesa (0.3)

     **they’re doing the operation again at the turn of the year (0.3)**

**36**     jalat on menny

     **my legs are gone**

**37 DR:  mm**

**38 EE:**   ei tuntua varpai[sa

     **no feeling in my [toes**

**39 SV:**            ** [mmh**

**40**    **(1.4)**

**41 EE:**   kaikki saat nii ni sitte veotaa johonki (.) tukeis- tämmöseen tuki

     **you get them all and then claim some musculo- this muscu-**

**42**    (0.6) [että tulleeha sit

     **(0.6) [so then you get**

**43 DR:**      [tules

           **[musculoskeletal diseases**

**44 EE:**  nii [tules             ni

    **yeah musculoskeletal diseases so**

**45 DR:**    [kyllä et kynnä mää panen ne sulle [kaikki ↑sinne

         **[sure I’ll put them all in for you [in ((in the application))**

**46 OSR:** **                        [mm**

**47 EE:**  pitäshän ne hakia koko paketti eihä sitä voi (0.4)

    **you should include the whole package surely you can’t (0.4)**

**48**    [tuollai

     **[like that**

**49 DR:** [niin no (.)

     **[yeah well (.)**

**50**     niin on ((nyökyttelee ja katsoo TT))

     **that’s right ((nodding and looking at EE))**

**51 EE:**  niin.

     **yeah.**

**52 DR:**  mutta sinulla on myös toimintakykyä.

    **but you do also have functional capacity.**

**53 NU:**  joo ja se [et niin

    **[yeah and that [so**

**54 DR:**           [et sinä oo niin[ko: vuodepotilas.

               **[you’re not like a bedridden patient.**

In this excerpt, the doctor sees the employee as having illnesses but still being capable of performing some work. By proposing a fixed-term partial sick leave (lines 3 and 4) instead of a partial disability pension for the current situation, she acknowledges the employee’s membership of the ill person category, treating him as being entitled to partial sick-leave. At the same time, she considers this membership insufficient for permanent inability to work, as fixed-term part-time sick leave is applied for when the employee is expected to eventually return to full-time work. By juxtaposing the employee’s age and the nature of his illness (“you’re at that age and the grounds for the application are musculoskeletal diseases” line 14) she implies that multiple factors, not just illnesses, must be considered when evaluating a person’s work ability.

Several aspects of the doctor’s proposal for fixed-term part-time sick leave indicate her anticipation of disagreement, aligning with the employee’s previously expressed desire to apply for partial disability pension. First, the doctor’s use of contrasting expressions (“but” on line 1 and “however” on line 4) implies she is proposing something else that the employee expects. Second, the design of her proposal constructs her as being on the same side as the employee (“we” lines 3 and 7). Third, after her proposal, she continues by warning the employee of a possible rejection of partial disability pension from the pension provider (lines 12, 14–16, and 18–19) and begins with an epistemic stance marker, “you know” (line 12), as a resource to deal with a potential disagreement (Asmuß, 2011). Fourth, she distances herself from the pension provider’s decisions (line 23) by mentioning the letters they “nowadays” send, creating neutrality and avoiding accountability (Potter, 1996).

The employee responds (from line 26 onward) to the doctor’s implication that his membership of the ill person category may not be sufficient to be granted part-time pension. His construction of the ill person category differs from that of the doctor, as he emphasizes the duration (“since nineteen seventy-eight,” lines 27–28), severity (“eyes have been operated on” in line 34, “my legs are gone” in line 36, “no feeling in my toes” in line 38), and the multiplicity (“you get them all,” line 41) of his illnesses. By providing a detailed description of his illness history, he legitimizes his claim for entitlement to a partial disability pension (see Potter, 1996).

The doctor aligns with the employee in considering his condition as a whole but continues to argue her perspective. Her response (line 45) shows that she is aware of the employee’s diabetes and its complications and intends to include them in the medical certificate for his partial disability pension application. However, she further constructs the employee as capable of working by stating that he still has some functional capacity, emphasizing her viewpoint by stressing the verb “do” (line 51) and using the expression “bedridden patient” (line 53).

In sum, the doctor considers the employee a member of the ill person category but also refers to other dimensions when constructing the employee’s work ability. The doctor’s perspective reflects a more modern conceptualization of work ability than that of the employee, who resists being categorized as a capable worker by using his illnesses as an argument. Thus, the meanings they construct for the ill person category are opposing, which leads to difficulties in the interaction.

## Discussion

In this study, we explored work ability as a social phenomenon that is constructed in multiparty encounters related to employees’ health and working life. We focused on how the negotiation participants construct and use descriptions of illness to explain and argue for and against work ability. Our study contributes to RTW research in three ways. First, it offers a new perspective of work ability: being realized during RTW negotiations through the participants’ interaction. Thus, work ability is talked into being (see Heritage and Clayman, 2010). Second, the study demonstrates how the participants use their cultural knowledge of illness and capability in their reasoning of work ability. Third, using MCA, the study shows how the situational nature of categories affects the participants’ shared understanding of the employee’s work ability and how these categories are consequential for the planning of employees’ future. Our findings provide important new insights into how shared understanding of the employee’s work ability is pursued in practice and how contemporary conceptualizations of illness and ability shape this process.

To our knowledge, MCA has not been previously applied in work ability research. Work ability has been studied from various perspectives, and as described in the Introduction, has different conceptualizations in both research and practice. However, this knowledge must be put into practice when collaboratively discussing the employee’s work ability. In medical decision-making, it has been established that discourse fundamentally shapes medical activities and the concept of medical evidence, even though they are routinely understood as objective professional practices (Caronia et al., 2017; Måseide, 2006). Similarly, our study suggests that work ability is constitutively shaped by the discourses in the context of RTW meetings, which was revealed by the analysis of these interactions that used discursive methods such as CA-informed MCA.

In addition to enabling a discursive perspective, MCA enabled us to examine how cultural knowledge of illness and capability is a part of the RTW negotiation participants’ reasoning and shared understanding of work ability. This stems from the core idea of MCA: categorization practices are part of our routine sense-making activities based on cultural knowledge (Watson, 2015). From this perspective, RTW negotiation participants’ own categories of illness and work ability, along with their categorization practices, are central, rather than the analyst’s predefined categories. For RTW research, this represents an analytical shift from using pre-existing categories or tools to explain participants’ actions to making their own categorization work visible (see Watson, 2015).

Our study shows that referring to illness and using it for reasoning work ability is relevant for participants and a common practice in RTW negotiations. This finding might seem surprising since conventionally, health information should be minimized in RTW negotiations. However, RTW participants’ categorization work of referring to employees’ illnesses and treating them as relevant to their work ability is understandable for several reasons. First, illness has traditionally equaled work disability in our society. Categories store a great deal of cultural knowledge about society (Sacks, 1992), and our findings are in line with this notion. Additionally, an individual’s illness and disability still form the basis for decisions on work disability benefits (OECD, 2010), which are often part of RTW planning. Second, employees may need to provide illness and disability descriptions as explanations for their inability to fulfill work responsibilities. In contemporary Western societies, inability to work may be more discrediting than having an illness itself (Hansen et al., 2014). According to Goffman (1955), such discrediting attributes need to be managed during interaction to construct and maintain a coherent and meaningful view of the self. Illness provides a socially acceptable explanation for not being able to perform work tasks (Parsons, 1951). Thus, references to illness categories can be a resource in face-work (Weiste et al., 2018) when employees aim to present themselves as social individuals whose inability to work is not related to social security disability “cheating” (Hansen et al., 2014). Third, categories are integral to the contexts in which they are used, as they define how people act and simultaneously constitute these contexts (Watson, 2015). RTW negotiations after sickness absence take place partly in the healthcare setting and partly in the working life context. The participants usually include a doctor, whose patient the employee often is. The context closely resembles a healthcare encounter and may direct discussions toward illnesses.

Ill person categories were used to explain and argue for or against the employee’s work ability. The participants treated the employee’s illness as a jointly accepted background and starting point for negotiating RTW decisions. Our findings are in line with those of a previous study that showed that sick-listed individuals’ health and disability served as starting points for the discussions on work ability and RTW, and that medical assessments legitimized stakeholders’ perspectives on these issues (Seing et al., 2012). However, our study shows that RTW negotiation participants construct the employee’s work ability using illness descriptions in a very situational way, and that these constructions are not always straightforwardly accepted. Illness, or more specifically the lack thereof, was also used to reassure the participants of the employee’s capability to work. Loisel et al. (2005) demonstrated that reassuring the participants of an employee’s work ability facilitates stakeholder collaboration in occupational rehabilitation. The act of reassuring can also be seen as persuasion, a common method used by healthcare professionals to involve patients in SDM (Matthias et al., 2020). Constructing the employee as capable by referring to the absence of illness may be a meaningful way for a doctor to try to support RTW. However, emphasizing the lack of illnesses may imply that employees with illnesses are not capable of working, contradicting the modern views of work ability and reinforcing the older perception of work disability as a straightforward consequence of illness. This reasoning could also complicate reframing work ability if the employee later develops an illness.

Our study illustrates how MCA can reveal the situational and consequential nature of categories of illness and work ability. Categorizing is always a situated practice that is closely tied to the context and actions in which the categories are used (Watson, 2015). Even though categories contain shared cultural knowledge, they are constructed and used differently in different situations and by different individuals. The RTW negotiation context involves different stakeholder perspectives alongside the cultural knowledge of illness and work ability. Occupational health physicians must balance between the employees’ wishes and their entitlement to social insurance benefits, the latter being crucial for deciding on future actions concerning the employees’ careers. The employees’ perceptions of their illnesses and the impacts of them are significant for the RTW processes, and their perceived work ability predicts labor outcomes such as sickness absence (Ahlstrom et al., 2010; Shaw et al., 2002). The employer perspective involves economic considerations, which in turn are influenced by contemporary societal issues such as changing working conditions and flexible labor markets (Seing et al., 2015).

In RTW negotiations, stakeholders gather to plan an employee’s RTW using their cultural knowledge about illness and work ability as a resource and their own incentives as guidance to accomplish things in the negotiation interaction. Based on our data, the constructions of work ability in these negotiations often reflect more contemporary perspectives of work ability. Although a shared understanding of the employee’s situation is often constructed by discussing illnesses, the participants simultaneously orient toward jointly planning work modifications to enable RTW. Thus, the shared acceptance of the employee’s membership of the ill person category entitles them to modifications. However, differing stakeholders’ constructions of the ill person category can lead to conflict and hinder accomplishing joint decisions. For example, by resisting the relevance of an ill person category for social insurance or other benefits in RTW negotiation, as seen in our last excerpt, the doctor may unauthorize some of the traditional rights of “ill persons” (Jutel, 2009). The employee’s resistance shown in the excerpt to the doctor’s suggestions can be seen as an attempt to create opportunities for active participation in SDM (Koenig, 2011).

Our study has strengths and limitations. The small sample limits generalizability to all RTW negotiations. However, the reliability of the study was increased by the systematic method of analysis (Stokoe, 2012) and by the data consisting of good-quality video recordings by two cameras (Peräkylä, 2011). While video recording may have influenced the topics discussed, the impact on the analysis was likely minimal, as the participants needed to focus on their institutional task. By rigorously examining the sequences, we were able to demonstrate turn-by-turn what happens in RTW negotiations. Detailed analysis of interaction allows for reflection on current institutional RTW negotiation practices.

For practitioners, our findings demonstrate that illness is central in RTW negotiation communications. Although not discussing illness in RTW negotiation is generally recommended, our study implies that this can be challenging. We recommend that healthcare professionals acknowledge and transparently explain the different perspectives on work ability, when pursuing a shared understanding of an employee’s situation

Our study also highlights that the construction and relevance of employees’ illnesses to their work ability may vary considerably among different negotiations or participants. Practitioners should understand that work ability in RTW negotiations is a socially constructed phenomenon formed situationally in each interaction. Situationally constructed work ability reflects participants’ differing viewpoints, interests and contexts, as well as the various conceptualizations of work ability in contemporary society. Further research is needed to explore how employees’ illnesses are discussed in other contexts related to work ability, beyond RTW after sickness absence.
